# Performance of probable dementia classification in a European multi-country survey

**DOI:** 10.1038/s41598-024-56734-7

**Published:** 2024-03-20

**Authors:** Matthias Klee, Kenneth M. Langa, Anja K. Leist

**Affiliations:** 1 Institute for Research on Socio-Economic Inequality, University of Luxembourg, Esch-sur-Alzette, Luxembourg; 2 Department of Internal Medicine, University of Michigan, Ann Arbor, MI USA

**Keywords:** Alzheimer's disease, Epidemiology

## Abstract

Feasibility constraints limit availability of validated cognitive assessments in observational studies. Algorithm-based identification of ‘probable dementia’ is thus needed, but no algorithm developed so far has been applied in the European context. The present study sought to explore the usefulness of the Langa–Weir (LW) algorithm to detect ‘probable dementia’ while accounting for country-level variation in prevalence and potential underreporting of dementia. Data from 56 622 respondents of the Survey of Health, Ageing and Retirement in Europe (SHARE, 2017) aged 60 years and older with non-missing data were analyzed. Performance of LW was compared to a logistic regression, random forest and XGBoost classifier. Population-level ‘probable dementia’ prevalence was compared to estimates based on data from the Organisation for Economic Co-operation and Development. As such, application of the prevalence-specific LW algorithm, based on recall and limitations in instrumental activities of daily living, reduced underreporting from 61.0 (95% CI, 53.3–68.7%) to 30.4% (95% CI, 19.3–41.4%), outperforming tested machine learning algorithms. Performance in other domains of health and cognitive function was similar for participants classified ‘probable dementia’ and those self-reporting physician-diagnosis of dementia. Dementia classification algorithms can be adapted to cross-national cohort surveys such as SHARE and help reduce underreporting of dementia with a minimal predictor set.

## Introduction

The WHO considers dementia, a condition characterized by memory and other cognitive impairments severe enough to cause the loss of independent function, to be a public health priority as the syndrome represents one of the main causes of death and dependency among older people^1,2^. Dementia causes significant economic, health and social care burden for those living with dementia and their informal caregivers. The number of people affected by dementia is expected to increase in the coming decades^3^. Due to resource intensity of systematic dementia ascertainment in representative cohort studies, algorithmic classifications of dementia are needed to inform research and reduce potential underreporting.

Dementia classification algorithms determine participants’ dementia status based on cognitive tests or sociodemographic variables that are readily accessible in cohort surveys such as the U.S. Health and Retirement Study (HRS)^4–9^. Existing algorithms frequently rely on (regression-based) prediction models or composite scores with an a priori cutoff for classification. In general, score cutoff based approaches facilitate interpretation, primarily due to a lower number of indicators and straight forward computation compared with regression-based classification. Langa, Kabeto and Weir developed a widely applied and previously validated score cutoff based algorithm to infer ‘probable dementia’ (LW)^4,5,10^. However, established dementia classification algorithms have not been systematically tested in the European, cross-national context, yet^4,5^.

The Survey of Health, Ageing and Retirement in Europe (SHARE) is a sister study to HRS. Nevertheless, direct application of well-established dementia classification algorithms is hindered due to differences in assessment protocols. Furthermore, cutoffs are not directly transportable since sample demographics, cognitive performance, indicator-outcome relationships, or reporting styles may vary across countries^11–13^.

We sought to examine the potential of the LW classification to detect ‘probable dementia’ using a minimal predictor set, with the aim of compensating for underreporting of dementia in cohort studies in the European context. Thus, we investigate the performance of a range of algorithms to detect ‘probable dementia’ and to adjust for country-level variation in underreporting of dementia in SHARE^14^. For this purpose, we adapted the LW classification to available indicators in SHARE, defining country specific cutoffs. Performance was compared to a set of benchmark machine learning (ML) algorithms to test for possible improvements with larger predictor sets and higher model complexity, specifically, a weighted logistic regression model, a random forest and an XGBoost classifier^15^. Validity of classifications was assessed (a) on the population level by comparing country-specific (probable) dementia prevalence before and after application of the algorithms to projections based on data from the Organisation for Economic Co-operation and Development (OECD) and a population representative study in Israel, and (b) on the individual level by assessing performance of those classified ‘probable dementia’ in further domains of health and cognitive function^16,17^.

## Methods

### Study population and design

SHARE is a representative, multi-country cohort study with over 140,000 participants aged 50 years and biennial follow-up (2004–2021)^14,18–20^. Activities of the SHARE-European Research Infrastructure Consortium (SHARE-ERIC) related to human subjects research are guided by international research ethics principles such as the Respect Code of Practice for Socio-Economic Research (professional and ethical guidelines for the conduct of socio-economic research) and the ‘Declaration of Helsinki’ (a set of ethical principles regarding human experimentation developed for the medical community by the World Medical Association, last revised at the 64^th^ WMA Meeting held in Fortalezza/Brazil in October 2013). SHARE waves 4 and following were reviewed and approved by the Ethics Council of the Max Planck Society (https://share-eric.eu/data/faqs-support). The SHARE data collection procedures are subject to continuous ethics review.

We used data from SHARE wave 7 (2017–2019) due to its large sample size across 26 European countries and Israel^18^. Data from countries with at least five participants self-reporting physician-diagnosis of dementia were eligible for analyses. Although we further address class imbalance, a cutoff of five was enforced to avoid biases in performance comparisons emerging from countries with outlying, extremely low dementia case numbers. Participants aged 60 years and older, with non-missing data on relevant sociodemographic, health or cognitive items were included in our analytic data set. All participants provided informed consent.

### Self-report physician-diagnosis of dementia

Participants self-report physician-diagnosis of dementia with a single item question (‘Has a doctor ever told you that you had/Do you currently have Alzheimer's disease, dementia, […]’)^21^.

### Langa–Weir classification

The LW algorithm classifies participants based on their performance in cognitive tests or based on items characterizing participants’ cognitive status that are provided by proxy respondents^4,5^. Proxy respondents answer on behalf of the main respondent in case of physical or cognitive limitations. The LW algorithm classifies participants with three groups: ‘normal’, ‘probable dementia’ (‘demented’ in original LW) and ‘cognitive impairment without dementia’ (CIND, ‘cognitive impairment, not demented’ in original LW)^4,5,22^.

LW classifications for self-respondents are based on immediate (0–10) and delayed (0–10) recall, Serial 7’s (0–5) and backwards counting (0–2)^4,5^. LW classifications for proxy respondents are based on proxy-rated memory (0–4), interviewer-perceived quality of cognition (0–2) and five instrumental activities of daily living (IADL) (0–5)^4,5^.

Cutoffs were defined so that the prevalence of categories resulting from classification of the full HRS sample match the population-level prevalence of CIND and dementia identified in the population-representative Aging, Demographics, and Memory Study (ADAMS) (equipercentile equating)^4,5,22^.

### Adaptation of the Langa–Weir classification—items

Distinctive features of SHARE and HRS hamper direct application of LW to SHARE, despite similar assessment protocols. First, there are no proxy-reported cognitive function measures available in SHARE wave 7. Second, nine limitations with IADLs are available in SHARE wave 7, but self-reported. Third, the backwards counting task is not available in SHARE wave 7. Fourth, the Serial 7’s is only available for a subset of 12 countries and was thus only used to assess validity of classifications in SHARE wave 7.

To examine the impact of including self-reported IADLs in classification, two LW adaptations were derived. LW (Recall) is based on immediate (0–10) and delayed (0–10) recall. LW (Recall & IADL) is based on LW (Recall) and nine IADLs (0–9) (Supplementary Table S1).

### Adaptation of the Langa–Weir classification—cutoffs

With a smaller number of items, sum score ranges are narrower, and hence pre-established cutoffs prone to misclassification. This motivated updating cutoff definitions for SHARE.

First, since there is no measure for CIND, we defined cutoffs for classifying ‘probable dementia’, but not CIND. However, definition of cutoffs with the equipercentile equating approach is hampered in absence of neuropsychological assessment informing about representative prevalence of dementia in SHARE. We thus introduced externally validated prevalence estimates based on a national representative study in Israel and projections published by the OECD (based on data from the World Alzheimer Report 2015 and population structure estimates from the United Nations)^16,17^.

Second, comparison suggested varying degrees of underreporting across countries, defined as discrepancy in prevalence estimates based on OECD data and self-report physician-diagnosis of dementia in SHARE^16,17,23,24^. In example, some countries with similar dementia prevalence in SHARE vary in prevalence according to OECD data^16,17^. Moreover, cross-national differences in mean recall performance and the number of reported IADLs indicate that cutoffs need to be defined within countries^16,17,23^.

Consequently, two sets of cutoffs for LW (Recall) were defined, based on (1) percentiles reflecting prevalence estimates reported by the OECD (equipercentile approach) and (2) the 2.5th percentile. (1) reflects external information on country-level dementia prevalence^16,17^. (2) is in line with the average population weighted dementia prevalence across countries in SHARE (M = 2.2%) and reflects an outlier definition, two standard deviations below the mean (for a normally distributed variable). Scores below cutoffs (1) or (2) led to LW (Recall) classification ‘probable dementia’.

For LW (Recall & IADL) a naïve IADL cutoff was defined to reflect outliers, one and a half interquartile ranges above Q3. In countries with Q3 equal to zero the cutoff was set to 1. Scores above this cutoff led to LW (Recall & IADL) classification ‘probable dementia’ if LW (Recall) was classified ‘probable dementia’, too.

Consequently, two LW algorithms were specified, i.e., based on Recall (LW [Recall]), or Recall and IADLs (LW [Recall & IADL]) with two alternative cutoffs for Recall (1—prevalence based; LW [Recall]^P^ or 2—outlier based; LW [Recall]). For LW (Recall & IADL) and LW (Recall & IADL)^P^ the same naïve IADL cutoff was used, irrespective of the cutoff used for Recall.

### Benchmark prediction models

To examine performance of different specifications we compared the four LW algorithms with different sets of indicators (based on cognitive tests and IALDs) and cutoffs (based on prevalence or outlier definitions). Additionally, we compared the four LW algorithms to three functions commonly classified as ML algorithms: a logistic regression model (GLM), a random forest (RF) and an XGBoost classifier (XGB), both latter relaxing parametrical assumptions and allowing for non-linear higher-order interactions^15,25^. RF classifier aggregate information of individual decision trees, created with random subsets of predictors following the concept of bootstrapping^25^. XGBoost classifier are based on a sequential ensemble of individual decision trees used to minimize the prediction error in final data partitions^15,25^.

ML algorithms included immediate and delayed recall, individual activities of daily living (ADL)/IADLs, and sociodemographic indicators age, education (tertiary/upper secondary/lower secondary), and sex/gender (male/female). Additionally, interviewer-rated variables were included comprising provided reading assistance (yes/no), willingness to answer (good/bad), clarification/comprehension questions (6-step Likert Scale from Never to Always). In rare circumstances proxies that were present during the interview reported IADLs on behalf of (0.7% of full sample), or together with the respondent (1.7% of full sample). Information on the presence and type of proxy was thus included in ML algorithms (No, Partner, Relative, Helper/Other). The outcome (class) used for model training was self-report physician-diagnosis of dementia.

To address class imbalance (i.e., majority of participants without self-report physician-diagnosis of dementia), three training sets were defined, by random split (Split, 50:50), downsampling (DOWN) the majority class, or the synthetic minority oversampling technique (SMOTE)^26^. With SMOTE, new cases are created based on the k-nearest neighbors of the minority class^26^.

Hyperparameters of RF and XGB models were tuned using grid search in five-fold cross validation with the area under the receiver operating characteristic curve (AUC) as criterion for selection of the best specification. Sampling weights were derived for GLM based on the inverse of the country-specific prevalence (or 1 minus the country-specific prevalence) for the minority (or majority) class^16,17^.

Consequently, LW algorithms were compared to 3 (GLM, RF, XGB) × 3 (Split, DOWN, SMOTE) + 1 GLM (weighted) benchmark ML-based algorithms. We will only discuss GLM weighted, RF SMOTE and XGB SMOTE in the following sections.

### Statistical analysis

Descriptive characteristics of the three training sets (Split, DOWN, SMOTE) and test set were assessed at baseline with Student’s t-tests for continuous and Chi-squared tests for categorical characteristics.

Model performance for all specifications was assessed in the same test set. In a first step, ML-based algorithms were trained and cutoffs for LW were defined in the training set. Second, classifications for LW and ML-based algorithms were computed for the test set, and performance was assessed comparing self-report physician-diagnosis of dementia to ‘probable dementia’ with multiple indicators (e.g., AUC, F1, sensitivity, specificity).

Then, country level variation in population-weighted ‘probable dementia’ prevalence was compared to previously reported estimates. First, per-country prevalence estimates were plotted according to observed dementia status in SHARE and previously reported figures. Then, underreporting across countries when applying classification algorithms was computed. Underreporting for individual countries was calculated as denoted in (1). N refers to the number of people living with dementia according to either data source. N_SHARE_ is the number of dementia cases in the test set, based on the population-weighted prevalence according to each algorithm. N_OECD_ is the number of dementia cases in the test set, based on prevalence estimates reported by the OECD.

1 $$underreporting = 1 - (n_{{{\text{SHARE}}}} /n_{{{\text{OECD}}}})$$

Prevalence estimates were mapped, to explore geographical patterns. Mean values in further domains of health and cognitive function were compared in ‘probable dementia’ and self-reported physician-diagnosis of dementia to assess validity of classifications. Finally, performance metrics were stratified by country to inspect fairness of classifications. All analyses were performed in R version 4.2.0^27^.

### Ethics approval

SHARE-ERIC's activities related to human subjects research are guided by international research ethics principles such as the Respect Code of Practice for Socio-Economic Research (professional and ethical guidelines for the conduct of socio-economic research) and the 'Declaration of Helsinki' (a set of ethical principles regarding human experimentation developed for the medical community by the World Medical Association, last revised at the 64th WMA Meeting held in Fortalezza/Brazil in October 2013). The SHARE study is subject to continuous ethics review. During Waves 1–4, SHARE was reviewed and approved by the Ethics Committee of the University of Mannheim. Wave 4 and the continuation of the project were reviewed and approved by the Ethics Council of the Max Planck Society. In addition, the country implementations of SHARE were reviewed and approved by the respective ethics committees or institutional review boards whenever this was required. The numerous reviews covered all aspects of the SHARE study, including sub-projects and confirmed the project to be compliant with the relevant legal norms and that the project and its procedures agree with international ethical standards.

### Consent to participate

Informed consent was obtained from all individual participants included in the study.

## Results

**Figure 1:**
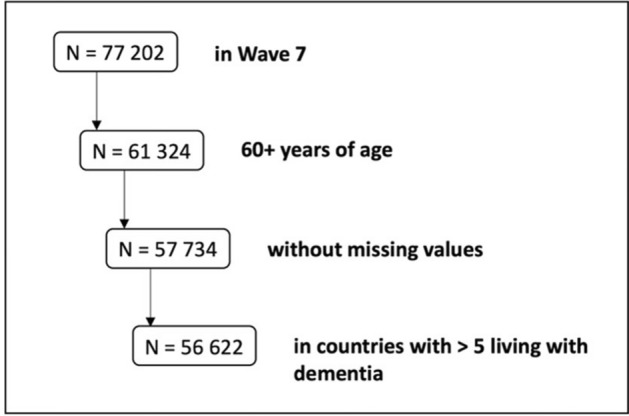
Flow chart illustrating sample size according to eligibility criteria.

Of 77 202 participants in SHARE wave 7, a total of 56 622 (M [SD] age, 71.7 [8.1] years, 56.3% female) from 26 countries were eligible to our analysis of which 2.1% reported physician-diagnosis of dementia (Fig. 1). Baseline characteristics are provided in Table 1.

**Table 1:** Descriptive characteristics of the training and test set.

	Test set	Training set		
	(*n* = 28 312)	Random split (*n* = 28,310)	DOWN (*n* = 1170)	SMOTE (*n* = 4095)
Age				
Mean (SD)	71.7 (8.05)	71.7 (8.08)	**75.4 (8.89)**	**73.9 (8.72)**
Gender				
Female	15 937 (56.3%)	15 931 (56.3%)	682 (58.3%)	2321 (56.7%)
Male	12 375 (43.7%)	12 379 (43.7%)	488 (41.7%)	1774 (43.3%)
Education (ISCED 1997)				
Lower secondary	11 418 (40.3%)	11 376 (40.2%)	**589 (50.3%)**	**1864 (45.5%)**
Upper secondary	9563 (33.8%)	9512 (33.6%)	**332 (28.4%)**	**1280 (31.3%)**
Tertiary	7331 (25.9%)	7422 (26.2%)	**249 (21.3%)**	**951 (23.2%)**
Dementia				
Yes	591 (2.1%)	585 (2.1%)	**585 (50.0%)**	**1170 (28.6%)**
No	27 721 (97.9%)	27 725 (97.9%)	**585 (50.0%)**	**2925 (71.4%)**

Reported *P*-values are based on Student’s t-tests for continuous and Chi-squared tests for categorical characteristics. Bold face illustrates variation across training and test set with *P* < .001. DOWN = downsampled training set. SMOTE = synthetic training set.

**Figure 2:**
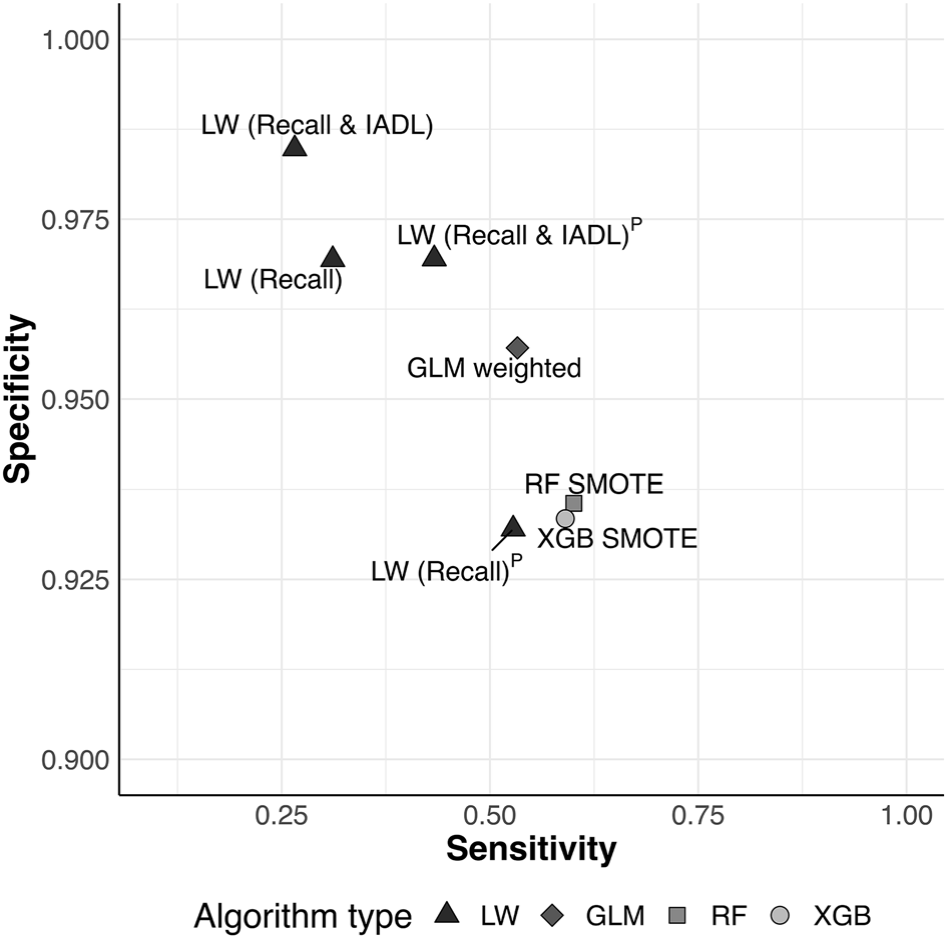
Sensitivity (x axis) against specificity (y axis) for Langa–Weir algorithms LW (Recall) with a Recall-cutoff reflecting the 2.5th percentile, LW (Recall)^P^ with a Recall-cutoff reflecting country-level dementia prevalence^16,17^, LW (Recall & IADL) based on LW (Recall) with an IADL cutoff reflecting 1.5 IQR above Q3, LW (Recall & IADL)^P^ based on LW (Recall)^P^ with an IADL cutoff reflecting 1.5 IQR above Q3, Logistic Regression (GLM weighted), Random Forest (RF SMOTE) and XGBoost (XGB SMOTE) algorithms in the test set.

Model performance was assessed regarding (balanced) accuracy, sensitivity, specificity (Fig. 2), precision, F1 and AUC^25^. All models accurately predicted ‘probable dementia’ (accuracy = 0.83–0.98). However, performance varied for metrics that are more robust in imbalanced data (balanced accuracy = 0.50–0.81; F1 = 0.01–0.30). Discrimination was moderate to good overall (AUC = 0.63–0.90). For LW, sensitivity was higher with prevalence-based compared to statistically informed Recall cutoffs (Supplementary Table S2). IADL inclusion in LW (Recall & IADL)^P^ increased specificity and combined good balanced accuracy (0.70), moderate AUC (0.70) and the best F1 across all algorithms (0.30).

For ML-based algorithms, GLM (weighted), RF SMOTE and XGB SMOTE showed the best performance combining good balanced accuracy (0.75–0.77), good AUC (0.86–0.89) and the best F1 within their algorithm type (0.26–0.30).

**Figure 3:**
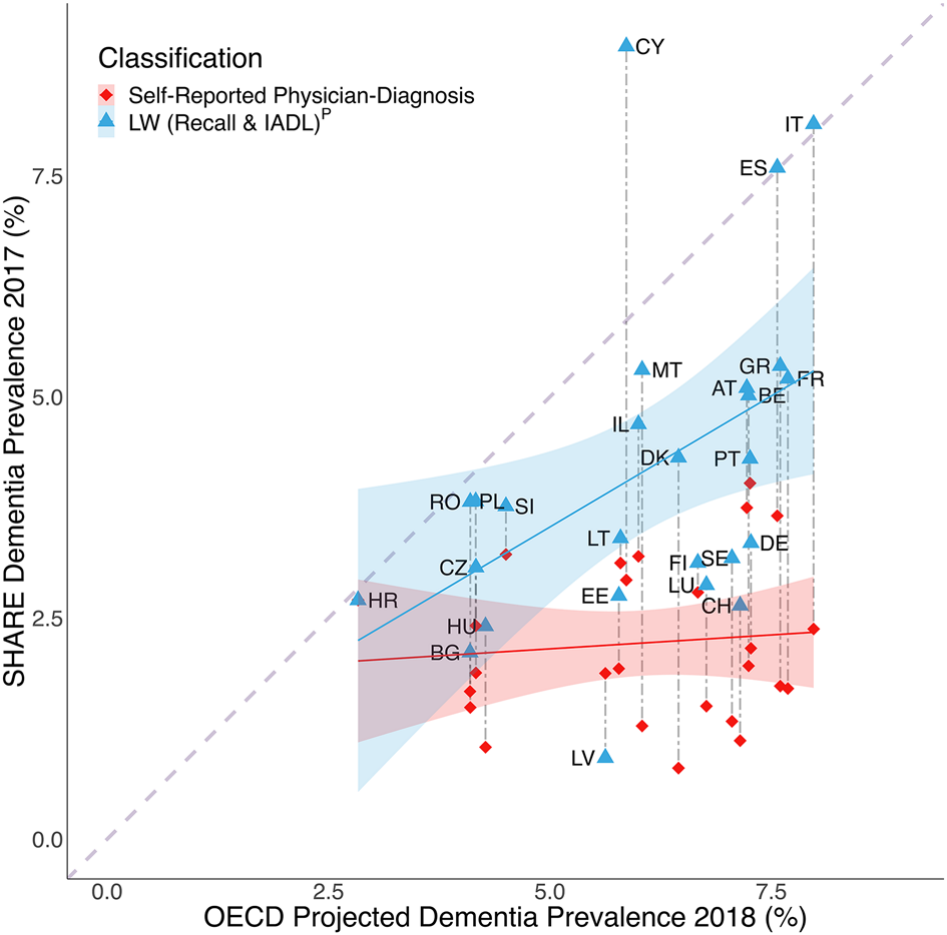
Dementia prevalence across countries. The y axis denotes population-weighted dementia prevalence observed in the Survey of Health, Ageing and Retirement in Europe (SHARE). The x axis denotes dementia prevalence based on projections from the Organisation for Economic Co-operation and Development (OECD) and a population-based study in Israel^16,17^. Red rectangles reflect country-level prevalence estimates for observed self-reported physician-diagnosis of dementia. Blue triangles reflect classified ‘probable dementia’ based on Langa–Weir (LW [Recall & IADL])^P^ with a Recall-cutoff reflecting country-level dementia prevalence and an IADL cutoff reflecting 1.5 IQR above Q3. Countries are coded according to ISO alpha 2 country code with labels next to blue triangles (Supplementary Table S3). Vertical dotted lines reflect the discrepancy between observed dementia prevalence in SHARE based on either self-reported physician-diagnosis of dementia or LW [Recall & IADL])^P^. The dotted diagonal reflects perfect overlap of dementia prevalence estimates based on SHARE and OECD. Shaded areas reflect confidence limits of linear models for self-report physician-diagnosis of dementia (red) or Langa–Weir (LW [Recall & IADL])^P^ (blue). Solid lines reflect linear models for dementia prevalence across countries with self-report physician-diagnosis of dementia (red) or Langa–Weir (LW [Recall & IADL])^P^ (blue). IADL = Instrumental Activities of Daily Living.

Regarding country-level variation in dementia prevalence, estimates based on SHARE with self-reported physician-diagnosis of dementia, or ‘probable dementia’ were compared to earlier reported country-specific prevalence (Fig. 3). LW (Recall & IADL)^P^ ‘probable dementia’ prevalence was more similar to previous findings, suggesting less underreporting. A steeper slope of the linear fit further suggests less variation in underreporting across countries.

**Figure 4:**
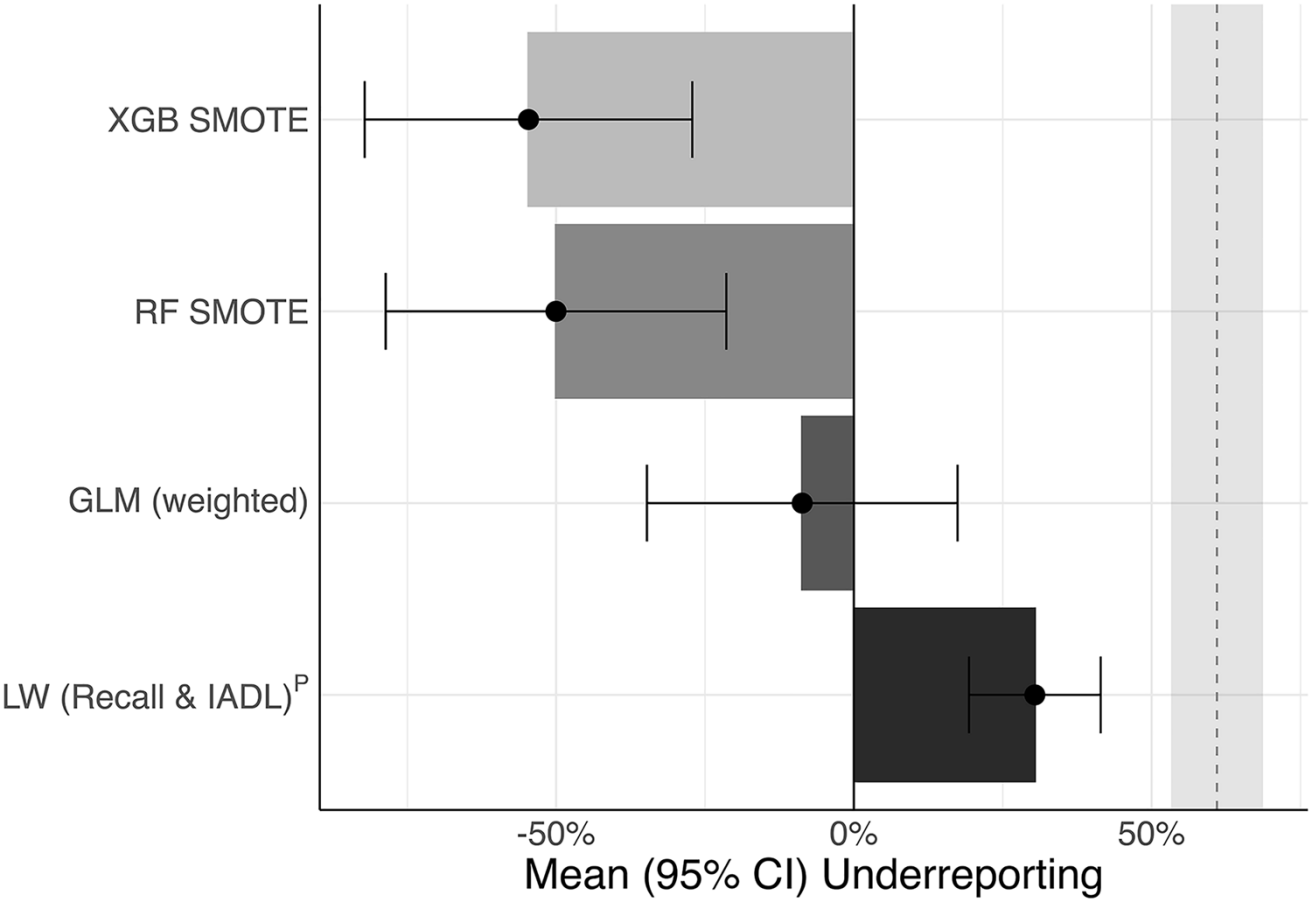
Mean underreporting and 95% CI across countries. Solid vertical line indicates perfect overlap of the number of people living with ‘probable dementia’ in the test set when applying classification algorithms and the number of people living with dementia in the test set calculated based on previously reported population-level prevalence estimates^16,17^. The dashed vertical line indicates mean underreporting (light grey fill indicates 95% CI) when the number of people living with self-reported physician-diagnosis of dementia is compared to previously reported estimates. Points indicate mean underreporting (dark fill indicates 95% CI) when the number of people living with ‘probable dementia’ is compared to previously reported estimates. LW (Recall)^P^ Langa–Weir algorithm with a Recall-cutoff reflecting country-level dementia prevalence and an IADL cutoff reflecting 1.5 IQR above Q3^16,17^. Logistic Regression (GLM weighted), Random Forest (RF SMOTE) and XGBoost (XGB SMOTE).

Underreporting with a prevalence estimate based on self-report physician-diagnosis of dementia was 61.0% (95% CI, 53.3–68.7%) on average. Underreporting with a prevalence estimate based on LW (Recall & IADL)^P^ ‘probable dementia’ was reduced to 30.4% (95% CI, 19.3–41.4%) on average (Fig. 4, Supplementary Table S3).

Prevalence estimates based on GLM (weighted) suggested higher variation in underreporting and a negative linear trend (results not shown) despite a better reduction in underreporting (mean [95% CI] underreporting = − 8.7% [− 34.8–17.4%]). Other ML algorithms drastically overestimated prevalence.

**Figure 5:**
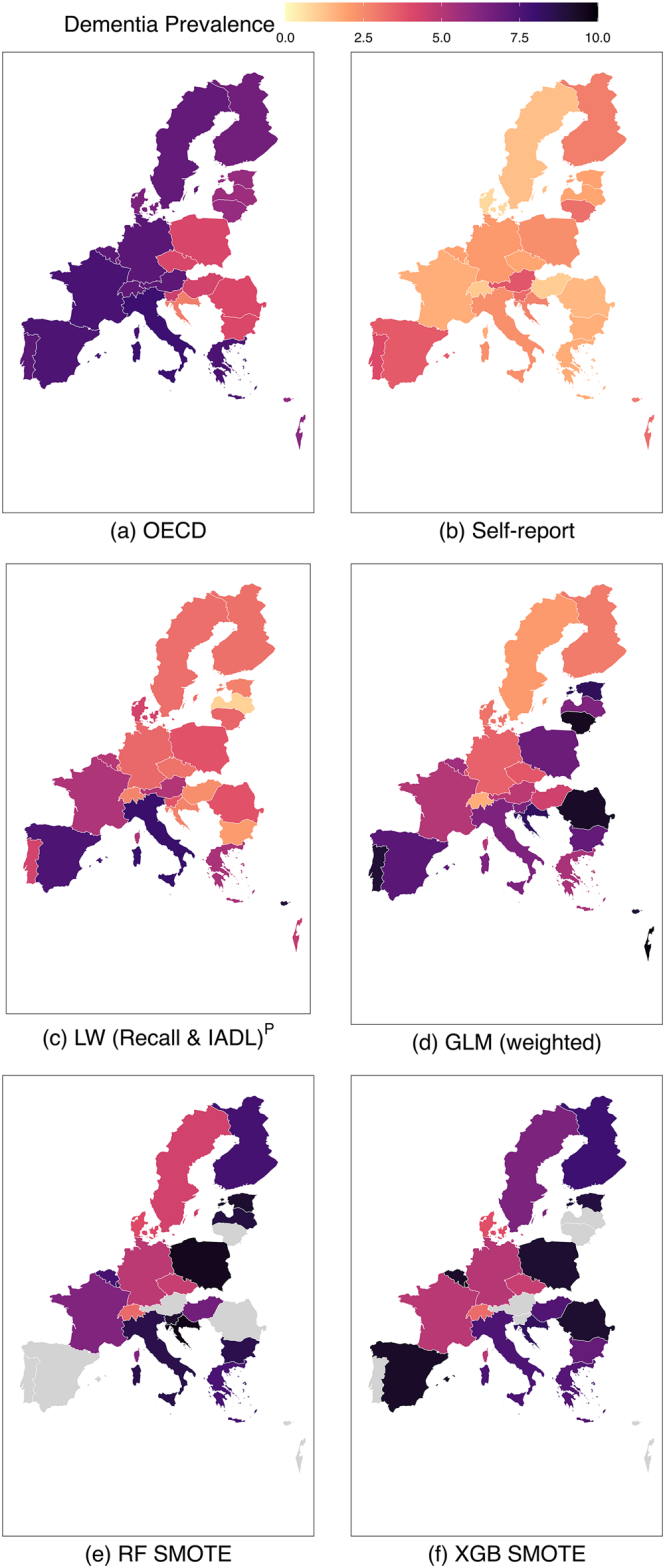
Population weighted dementia prevalence across countries based on data from the Organisation for Economic Co-operation and Development (OECD) and a nationally representative study in Israel, self-reported physician-diagnosis or ‘probable dementia’ classification. Grey fill indicates prevalence > 10%. LW (Recall)^P^ Langa–Weir algorithm with a Recall-cutoff reflecting country-level dementia prevalence and an IADL cutoff reflecting 1.5 IQR above Q3^16,17^. Logistic Regression (GLM weighted), Random Forest (RF SMOTE) and XGBoost (XGB SMOTE).

Prevalence estimates were further mapped to explore geographical patterns (Fig. 5). Whereas previously reported estimates and SHARE estimates based on self-reported physician-diagnosis of dementia suggested overall differences in magnitude, previously reported estimates indicated low variation between neighboring countries. Prevalence was overall higher with LW (Recall & IADL)^P^ ‘probable dementia’ compared to self-reported physician-diagnosis of dementia but lower compared to OECD projections. Differences in prevalence between neighboring countries were smaller with LW (Recall & IADL)^P^ ‘probable dementia’ compared to self-reported physician-diagnosis of dementia but higher compared to previously reported estimates. GLM (weighted), RF SMOTE and XGB SMOTE reinforced discrepancies between some neighboring countries and exceeded previously reported prevalence estimates.

**Figure 6:**
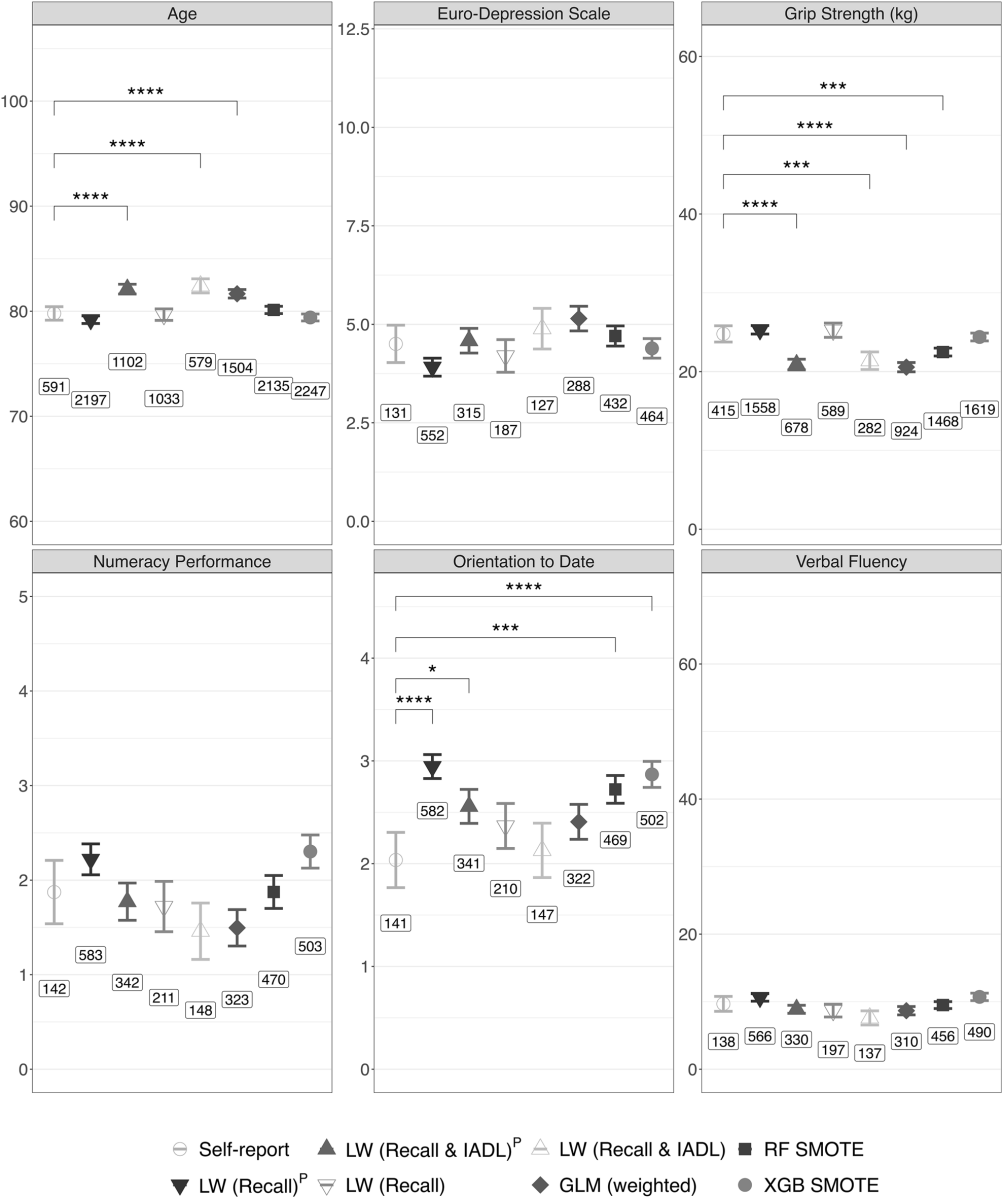
Validity assessment in further domains of health and cognitive function; means with 95% CIs for algorithmic ‘probable dementia’ and self-reported physician-diagnosis of dementia; brackets indicate Bonferroni-adjusted p values from Student’s t-tests; ****p < .0001 ***p < .001 **p < .01 *p < .05; Euro-Depression scale ranges from 0 to 12 with higher values indicating higher burden of depressive symptoms; numeracy performance relates to Serial 7’s task; orientation to date relates to 4 questions about the current date. Labels refer to group sizes, i.e., participants with ‘probable dementia’ or self-report physician-diagnosis of dementia and non-missing data. Note that Euro-Depression scale, numeracy performance, orientation to date and verbal fluency were only available for a subset of 12 countries. LW (Recall) with a Recall-cutoff reflecting the 2.5th percentile, LW (Recall)^P^ with a Recall-cutoff reflecting country-level dementia prevalence^16,17^, LW (Recall & IADL) based on LW (Recall) with an IADL cutoff reflecting 1.5 IQR above Q3, LW (Recall & IADL)^P^ based on LW (Recall)^P^ with an IADL cutoff reflecting 1.5 IQR above Q3, Logistic Regression (GLM weighted), Random Forest (RF SMOTE) and XGBoost (XGB SMOTE).

Validity was further assessed comparing mean values in further domains of health and cognitive function between ‘probable dementia’ and self-reported physician-diagnosis of dementia in complete cases (Fig. 6). Results suggest good fit overall for depressive symptoms, verbal fluency, and numeracy performance^28^. Grip strength aligned best with LW (Recall), LW (Recall)^P^, and XGB SMOTE, just like age. Regarding orientation to date, only LW ‘probable dementia’ algorithms with statistically informed Recall cutoffs and GLM (weighted) overlap with self-reported physician-diagnosis of dementia.

Performance metrics were stratified by country to inspect fairness of algorithms (Supplementary Fig. S4). Variation in performance is higher for LW (Recall & IADL)^P^ compared to ML-based algorithms for AUC, F1, precision, and sensitivity, but for balanced accuracy, variation is similar. For accuracy and specificity LW (Recall & IADL)^P^ shows the least variation.

## Discussion

In this study, we adapted the LW dementia classification algorithm and tested its ability to detect ‘probable dementia’ in the European context. LW proved useful to detect ‘probable dementia’ compared to when classification is based entirely on self-report of a physician-diagnosis. In validity checks on the population level, we found that LW based on immediate recall, delayed recall and IADLs with a prevalence-based recall cutoff (LW [Recall & IADL]^P^) performed best in reducing underreporting across countries. On the individual level, performance profiles in other domains of health and cognitive function such as numeracy in ‘probable dementia’ matched those in participants who self-report physician-diagnosis of dementia in a subset of countries. Despite higher complexity and a larger number of indicators, ML-based classifiers performed less consistent across countries reinforcing the superiority of the adapted LW classification to help identify ‘probable dementia’ with a minimal predictor set.

A previous study suggested validity of LW classifications in the US context and performance in line with algorithms additionally incorporating demographic characteristics^10^. We found similar sensitivity and high specificity of the adapted LW (Recall & IADL)^P^ in the European context, despite a smaller set of indicators^10^. More recently, other ML-based algorithms classifying ‘probable dementia’ were evaluated in the European context^29–32^. In line with our findings, a recent study suggested limited surplus in performance over logistic regression models when using complex ML-based algorithms, and only so with survival analyses^29^. As an alternative to supervised learning, where models are trained on an a priori labelled class, a recent study applied a previously established unsupervised ML approach to clustering in SHARE, longitudinally^30,32^. AUC and sensitivity of LW (Recall & IADL)^P^ classification were in line with the clustering based classification in SHARE wave 7, although being marginally lower^30^. This suggests similar classification performance with the score cutoff based algorithm. LW (Recall & IADL)^P^ application requires no longitudinal follow-up and hence, compared with longitudinal algorithms, we were able to classify more data points from more countries at a given wave, which however precluded direct comparison, e.g., of the number of newly identified ‘probable dementia’^30^. Nonetheless, including external information on population-level dementia prevalence suggested that LW (Recall & IADL)^P^ identified an expected additional number of ‘probable dementia’, thus suggesting reduced potential underreporting, while maintaining high specificity^16,17^.

We found variation in classification performance across countries. Such variation was also apparent in a recent study in SHARE, and may be due to descriptive differences across countries, emerging e.g., from differences in population structures^30^. As an example, sensitivity was lowest in countries with lower mean recall performance, especially with LW (Recall & IADL)^P^, suggesting floor effects during cutoff definitions. Furthermore, prevalence in countries with distinct distributions of antecedents to dementia, was systematically overestimated with ML-based classifiers (e.g., Eastern compared to Northern European countries)^33^. Another reason for country-level variation may be differential association of included indicators with dementia risk, e.g., depending on welfare regimes, or policy across countries. Although we cannot rule out that emerging biases reduce performance for some countries, inspection of performance metrics when excluding data from one country at a time during training and testing did not alter main findings (results not shown).

Compared with benchmark ML-based algorithms, LW (Recall & IADL)^P^ suggested higher and more consistent specificity across countries. When using ML-based classifiers such as RF/XGB SMOTE to detect ‘probable dementia’, our results suggest a lack of consistency in prevalence estimates of neighboring countries. More dramatically, ML-based ‘probable dementia’ prevalence exceeded population-based projections and GLM (weighted) introduced a negative association between SHARE-based prevalence estimates and those informed by previous findings^16,17,23^. Contrary, with LW (Recall & IADL)^P^, prevalence in neighboring countries was more homogeneous and more similar to previously reported estimates, leading to a positive association between prevalence estimates based on SHARE and previously reported estimates.

Critically, projected prevalence estimates used to assess validity of classifications come with considerable uncertainty stemming from oversimplification (e.g., assuming constant age-specific prevalence), varying operationalizations or lacking knowledge about future developments in medicine or policy^34^. Further, estimates of the OECD reflect projections for 2018 based on data from 2015. However, time lag was low and OECD prevalence estimates were generally higher than those based on self-report physician-diagnosis in SHARE. Still, prevalence may be understated due to healthy volunteer bias, or lacking representativeness in underlying studies, or systematic underdiagnosis in low- and middle-income countries^31^. Critically, receiving a diagnosis given dementia may depend on the severity of symptoms, lacking access to screening tools, or lacking knowledge of or access to treatment and care^11^. Moreover, stigma evolving around dementia may result in longer times until diagnosis, with apparent variation in such stigma across European countries, aligning with the availability of specialized care^35^. In any case, self-reporting a dementia diagnosis may amplify such biases^36^. It is crucial to interpret our findings acknowledging absence of a gold-standard measure of dementia prevalence and thus discrepancies between the number of people living with dementia with or without a diagnosis to self-report. As such, we refer to underreporting resulting from multiple processes encompassing but not limited to failure to self-report a present diagnosis or absence of a diagnosis despite presence of dementia. In absence of clinically valid assessment of dementia inclusion of external information allowed employing the equipercentile equating approach to SHARE and a consequent exploration of mechanisms leading to differences between data sources^4,5^. As such, our findings suggest that LW (Recall & IADL)^P^ efficiently reduced underreporting defined as discrepancy between previously reported estimates and SHARE-based estimates, uniformly across countries.

Internal validation further suggested that LW (Recall & IADL)^P^ ‘probable dementia’ was similar to self-reported physician-diagnosis of dementia regarding further domains of health and cognitive function in a subset of countries with available markers. GLM (weighted), and LW adaptations including IADLs overstated age and understated grip strength, both reflecting risk factors of dementia^37^. Our findings suggest these algorithms classify older, physically more impaired participants irrespective of potentially underlying or absent dementia thus increasing noise and deteriorating fairness with respect to ageism. Inclusion of IADLs, conveying information on worsening physical health but not dementia, specifically, may explain this. Interestingly, IADL inclusion had a positive effect on specificity, possibly by accounting for floor effects in recall measures. Whereas XGB SMOTE and GLM (weighted) ‘probable dementia’ fit well to self-reported physician-based dementia diagnosis, prevalence estimates were highly overstated with XGB SMOTE, and biased across countries with GLM (weighted). Our findings further suggest calibration may be negatively affected in algorithms trained with SMOTE^38^. Verbal fluency, depressive symptoms, and numeracy performance were similar in self-report physician-diagnosis of dementia and ‘probable dementia’ across algorithms^4,5,39^. Critically, depressive symptoms may play a role as early sign or risk factor of dementia, or relate to recall performance (r = − 0.28, *P* < 0.001) and IADL reporting (r = 0.37, *P* < 0.001) irrespective of dementia^40,41^. Orientation to date was not well reflected by LW classifications with a prevalence-based recall cutoff, or RF/XGB SMOTE, potentially due to the categorical operationalization. In sum, our results support similarity of ‘probable dementia’ and self-reported physician-diagnosis of dementia in most algorithms.

This study systematically investigated a range of dementia classification algorithms to adjust for underreporting of dementia in a large European ageing survey, using internally derived and externally validated prevalence estimates. Some limitations need to be considered when interpreting our findings. First, we could not train models on CIND classification and thus participants with mild limitations may be misclassified ‘without probable dementia’. Second, dementia rates were lower in our sample than in previous studies reducing statistical power to detect ‘probable dementia’. Further, a smaller number of participants self-reporting dementia limits generalizability of the validation procedure^10^. Third, discrepancies in dementia prevalence which we interpreted as potential underreporting may be due to selection bias, or due to diagnoses being based on self-reports, both of which could lead to misclassification following stricter cutoff definitions. Related, models trained on self-reported physician-diagnosis of dementia, which is less reliable than formal diagnosis, may miss prevalent cases due to reduced statistical power during training^42^. Fourth, participants self-reporting limitations may systematically differ from those not disclosing such information impeding generalizability of our findings^43^. Fifth, LW was adapted to a reduced set of indicators and self-reported IADLs reducing discriminatory power. However, recall scores contributed most to LW (20/27 points) and mean Serial 7’s scores for LW (Recall & IADL)^P^ ‘probable dementia’ and self-report physician-diagnosed dementia did not differ significantly in a subset of the data, suggesting limited added value of including Serial 7’s for classification. Sixth, a previous study suggested the need for model stratification^44^. However, class imbalance, sample size and lacking diversity prohibited fairness evaluation of classifications in stratified samples. Seventh, discussed algorithms were applied to cross-sectional data, and may misclassify participants with outlying low performance. Further, LW (Recall & IADL)^P^ cannot differentiate prevalent or incident ‘probable dementia’. Eighth, participants in our study were younger (age 60 and older) compared to ADAMS (age 70 and older), likely healthier (complete case, community-dwelling) and proxy-ratings were not available, which potentially reduced power to detect cases and yielded more conservative cutoffs^22^. We thus call for the inclusion of proxy assessments to bolster research relating to cognitive ageing and dementia.

In absence of clinically validated dementia assessment in observational studies, classification algorithms such as LW can be adapted to cross-national cohort surveys such as SHARE to reduce underreporting of dementia. In this study, LW (Recall & IADL)^P^ identified ‘probable dementia’ with high validity compared to ML-based classifiers. Many large ageing surveys provide recall items or IADLs^45,46^. We thus provide a transparent and transportable classification with a minimal predictor set, based on the pre-established LW algorithm. While ‘probable dementia’ does not reflect a diagnosis, we hope to empower dementia researchers in several ways. First, the present work may facilitate uptake of dementia classification algorithms for research in SHARE. Additionally, we provide knowledge to transport classifications into other applications, since cutoffs used for classification are directly interpretable and adaptable across settings. Second, classifications may be used to inform sampling strategies. Finally, a ‘probable dementia’ indicator may improve statistical power, offering means to assess sensitivity in a multitude of research applications. Future research may offer opportunities to validate our findings with the Harmonized Cognitive Assessment Protocol (HCAP) and compare performance across sister studies of SHARE and HRS^46^. This may also yield the potential to investigate algorithm performance in subgroups for fairness evaluations and disparities research.

### Supplementary Information

Supplementary Information.

## Data Availability

The data underlying this article are available in [The Survey of Health, Ageing and Retirement in Europe (SHARE)], at https://doi.org/10.6103/SHARE.w7.800. The datasets were derived from sources in the public domain: [SHARE, www.share-project.org].

## Code availability

Code is available at https://github.com/makleelux/probable_dementia_SHARE.
