# Supplementary material for: Performance of probable dementia classification in a European multi-country survey
Source: Sci Rep. 2024 Mar 20;14:6657. doi: 10.1038/s41598-024-56734-7 (PMC10954769; doi:10.1038/s41598-024-56734-7)
Supplement: Supplementary file 1 — Supplementary Information. [file 41598_2024_56734_MOESM1_ESM.docx]

# Supplementary Material

M. Klee^1^, K. M. Langa^2^, A. K. Leist^1^*. (Submitted to Scientific Reports) Performance of Probable Dementia Classification in a European Multi-country Survey.

^1^Institute for Research on Socio-Economic Inequality, University of Luxembourg, Esch-sur-Alzette, Luxembourg.
^2^Department of Internal Medicine, University of Michigan, Ann Arbor, MI, USA.

*Corresponding author(s).
E-mail(s): anja.leist@uni.lu

**Supplementary Table S1.** Comparison of items constituting LW adaptations.

| Characteristics | HRS | (Recall) | (Recall & IADL) |
| --- | --- | --- | --- |
| Cognitive Function |  |  |  |
| Immediate Recall | SR | SR | SR |
| Delayed Recall | SR | SR | SR |
| Serial 7’s | SR | - | - |
| Backward Counting | SR | - | - |
| IADL |  |  |  |
| Preparing Meals | Proxy | - | SR |
| Shopping Groceries | Proxy | - | SR |
| Making Phone Calls | Proxy | - | SR |
| Taking Medication | Proxy | - | SR |
| Managing Money | Proxy | - | SR |
| Using a Map | - | - | SR |
| Doing Housework | - | - | SR |
| Independent Mobility | - | - | SR |
| Doing Laundry | - | - | SR |

Proxy = Proxy respondent. SR = Self-reported.

**Supplementary Table S2.** Performance of algorithmic classifications in the test set.

| Classification | Accuracy | Balanced Accuracy | Sensitivity | Specificity | Precision | F1 | AUC |
| --- | --- | --- | --- | --- | --- | --- | --- |
| LW (Recall) | 0.96 | 0.64 | 0.31 | 0.97 | 0.18 | 0.23 | 0.64 |
| LW (Recall)^P^ | 0.92 | 0.73 | 0.53 | 0.93 | 0.14 | 0.22 | 0.73 |
| LW (Recall & IADL) | 0.97 | 0.63 | 0.27 | 0.98 | 0.27 | 0.27 | 0.63 |
| LW (Recall & IADL)^P^ | 0.96 | 0.70 | 0.43 | 0.97 | 0.23 | 0.30 | 0.70 |
| GLM | 0.98 | 0.55 | 0.11 | 1.00 | 0.51 | 0.18 | 0.88 |
| GLM weighted | 0.95 | 0.75 | 0.53 | 0.96 | 0.21 | 0.30 | 0.89 |
| GLM DOWN | 0.84 | 0.80 | 0.76 | 0.84 | 0.09 | 0.17 | 0.88 |
| GLM SMOTE | 0.93 | 0.77 | 0.60 | 0.94 | 0.16 | 0.26 | 0.88 |
| RF | 0.98 | 0.52 | 0.04 | 1.00 | 0.70 | 0.07 | 0.90 |
| RF DOWN | 0.84 | 0.81 | 0.77 | 0.84 | 0.10 | 0.17 | 0.89 |
| RF SMOTE | 0.93 | 0.77 | 0.60 | 0.94 | 0.17 | 0.26 | 0.88 |
| XGB | 0.98 | 0.50 | 0.00 | 1.00 | 1.00 | 0.01 | 0.89 |
| XGB DOWN | 0.83 | 0.80 | 0.77 | 0.84 | 0.09 | 0.16 | 0.88 |
| XGB SMOTE | 0.93 | 0.77 | 0.62 | 0.93 | 0.16 | 0.26 | 0.86 |

^P^ = prevalence-based cutoff. GLM = Logistic Regression. RF = Random Forest. XGB = XGBoost.

**Supplementary Table S3.** Dementia prevalence and number of expected dementia cases across algorithms.

|  |  |  | OECD | | SR-PD | | LW (R&I)^P^ | | GLM (weighted) | | RF SMOTE | | XGB SMOTE | |
| --- | --- | --- | --- | --- | --- | --- | --- | --- | --- | --- | --- | --- | --- | --- |
| Country | ISO | n | Prev. | n | Prev. | n | Prev. | n | Prev. | n | Prev. | n | Prev. | n |
| Austria | AT | 1,267 | 7.22 | 91.52 | 3.75 | 47.47 | 5.10 | 64.63 | 4.89 | 61.99 | 10.11 | 128.05 | 14.37 | 182.04 |
| Belgium | BE | 1,791 | 7.24 | 129.73 | 1.96 | 35.08 | 5.02 | 89.85 | 5.57 | 99.73 | 7.74 | 138.69 | 9.32 | 166.96 |
| Bulgaria | BG | 700 | 4.10 | 28.68 | 1.67 | 11.69 | 2.11 | 14.77 | 7.07 | 49.49 | 8.61 | 60.24 | 7.02 | 49.13 |
| Croatia | HR | 860 | 2.83 | 24.37 | 2.70 | 23.25 | 2.70 | 23.24 | 8.33 | 71.61 | 9.71 | 83.48 | 8.35 | 71.81 |
| Cyprus | CY | 467 | 5.86 | 27.38 | 2.93 | 13.68 | 8.96 | 41.86 | 9.06 | 42.31 | 11.87 | 55.44 | 14.70 | 68.67 |
| Czech Rep. | CZ | 1,774 | 4.16 | 73.87 | 1.88 | 33.35 | 3.07 | 54.49 | 3.73 | 66.09 | 4.60 | 81.67 | 4.59 | 81.47 |
| Denmark | DK | 1,164 | 6.45 | 75.10 | 0.80 | 9.35 | 4.31 | 50.22 | 3.07 | 35.75 | 4.01 | 46.69 | 3.91 | 45.48 |
| Estonia | EE | 1,864 | 5.78 | 107.71 | 1.93 | 35.93 | 2.75 | 51.30 | 8.39 | 156.39 | 9.22 | 171.89 | 8.88 | 165.53 |
| Finland | FI | 680 | 6.67 | 45.35 | 2.79 | 18.97 | 3.12 | 21.25 | 2.86 | 19.44 | 7.75 | 52.73 | 7.93 | 53.93 |
| France | FR | 1,307 | 7.69 | 100.46 | 1.70 | 22.25 | 5.21 | 68.05 | 5.07 | 66.24 | 6.31 | 82.53 | 4.93 | 64.40 |
| Germany | DE | 1,406 | 7.27 | 102.21 | 2.16 | 30.33 | 3.35 | 47.08 | 3.47 | 48.82 | 4.89 | 68.74 | 5.03 | 70.72 |
| Greece | GR | 1,263 | 7.60 | 96.00 | 1.73 | 21.86 | 5.35 | 67.57 | 5.33 | 67.3 | 7.69 | 97.13 | 7.40 | 93.44 |
| Hungary | HU | 646 | 4.27 | 27.60 | 1.04 | 6.72 | 2.41 | 15.55 | 4.40 | 28.44 | 6.67 | 43.08 | 7.48 | 48.33 |
| Israel | IL | 821 | 6.00 | 49.26 | 3.20 | 26.25 | 4.69 | 38.52 | 9.83 | 80.67 | 11.22 | 92.10 | 11.47 | 94.16 |
| Italy | IT | 1,710 | 7.98 | 136.41 | 2.38 | 40.63 | 8.08 | 138.24 | 6.39 | 109.2 | 8.66 | 148.03 | 7.59 | 129.76 |
| Latvia | LV | 574 | 5.63 | 32.29 | 1.87 | 10.76 | 0.92 | 5.27 | 6.37 | 36.57 | 8.84 | 50.73 | 10.81 | 62.04 |
| Lithuania | LT | 668 | 5.80 | 38.72 | 3.12 | 20.85 | 3.40 | 22.73 | 9.52 | 63.58 | 11.85 | 79.17 | 14.01 | 93.59 |
| Luxembourg | LU | 404 | 6.77 | 27.34 | 1.50 | 6.07 | 2.87 | 11.61 | 3.96 | 16.01 | 8.23 | 33.23 | 9.79 | 39.56 |
| Malta | MT | 468 | 6.04 | 28.27 | 1.28 | 5.99 | 5.31 | 24.83 | 3.48 | 16.28 | 6.36 | 29.76 | 7.48 | 35.02 |
| Poland | PL | 1,599 | 4.16 | 66.55 | 2.41 | 38.59 | 3.82 | 61.08 | 6.84 | 109.35 | 9.58 | 153.14 | 9.16 | 146.47 |
| Portugal | PT | 489 | 7.26 | 35.50 | 4.02 | 19.68 | 4.30 | 21.03 | 9.04 | 44.23 | 14.61 | 71.46 | 12.34 | 60.34 |
| Romania | RO | 714 | 4.10 | 29.28 | 1.49 | 10.63 | 3.81 | 27.23 | 9.33 | 66.64 | 11.35 | 81.02 | 9.19 | 65.62 |
| Slovenia | SI | 1,435 | 4.50 | 64.62 | 3.22 | 46.19 | 3.76 | 54.01 | 6.46 | 92.75 | 9.18 | 131.78 | 11.88 | 170.42 |
| Spain | ES | 1,827 | 7.57 | 138.25 | 3.66 | 66.78 | 7.59 | 138.70 | 7.28 | 132.96 | 10.59 | 193.48 | 9.35 | 170.81 |
| Sweden | SE | 1,407 | 7.06 | 99.28 | 1.33 | 18.74 | 3.18 | 44.69 | 2.16 | 30.36 | 4.32 | 60.85 | 6.44 | 90.57 |
| Switzerland | CH | 1,004 | 7.15 | 71.76 | 1.11 | 11.18 | 2.64 | 26.51 | 1.70 | 17.09 | 3.32 | 33.31 | 3.28 | 32.92 |
| Total |  | 28 309 |  | 1,747.51 |  | 632.27 |  | 1,224.31 |  | 1,629.29 |  | 2,268.40 |  | 2,353.18 |

Numbers are rounded to the second decimal. Population-weighted prevalence and number of cases based on the test set. Prev. = Prevalence. SR-PD = self-reported physician-diagnosis. LW (R & I)^P^ = Langa-Weir classification with Recall & IADLs and a prevalence-based cutoff. OECD = based on projections from the Organisation for Economic Co-operation and Development and a population-based study in Israel. Three participants excluded due to missing sampling weights.


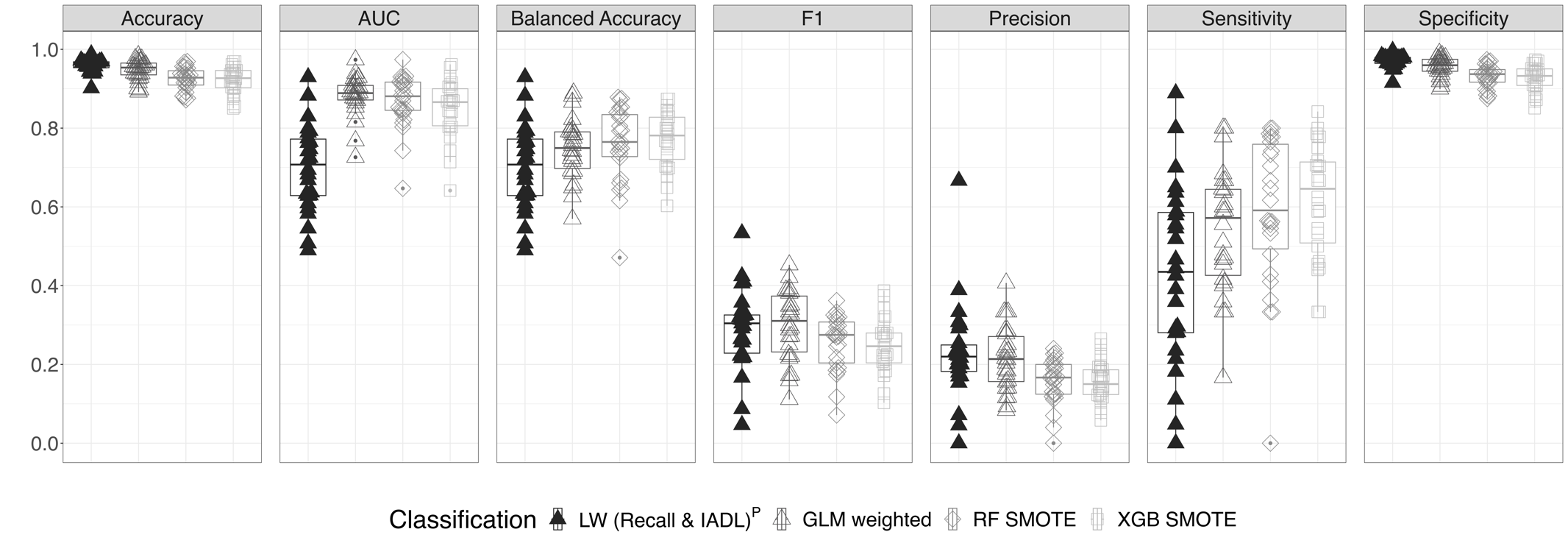


**Supplementary Fig. S4** Dispersion of algorithm performance in test data across countries; dots represent countries. ^P^ = prevalence-based cutoff
